# A Question on the Physiology of the Brain

**Published:** 1875-10

**Authors:** 


A Question on the Physiology of the Brain.—The present generation of medical men have been taught that lesions of either hemisphere of the brain communicate their results to the opposite side of the body. The decussation of the fibres at the base of the brain furnished a ready explanation of the phenomenon. But now this must be unlearned. Brown Sequard has collected 150 cases of paralysis in which the brain-lesion was on the paralyzed side. He says: "The character of the symptoms in brain-diseases is not in the least dependent on the seat of the lesion, so that a lesion of the same part may produce a great variety of symptoms, while on the other hand the symptoms may be due to the most various causes—various not only as regards the kind, but also the seat of the organic alteration. In view of these facts," he continues, "I have been led to believe that lesions of the brain produce symptoms, not by destroying the function of the part where they exist, but by exerting over distant parts an inhibitory or an exciting influence, or in other words, either by stopping an activity or setting it in play." These views are in direct opposition to the doctrines of phrenology.—*Pacific Med. and Surg. Journal.*

